# Water Supply and Typhoid Fever

**Published:** 1897-06-12

**Authors:** 


### Water Supply and Typhoid Fever.

In an article on water supply and typhoid fever which appeared in the *Times* on June 7th, attention was drawn to the probability of typhoid fever being distributed by other means than water, which of course is generally admitted by authorities on the subject. When, however, the *Times* goes on to state that Dr. Seaton has prepared a map of Surrey which shows in regard to every part of the county the source of its public water supply, and when the hope is expressed that by plotting out upon this all the cases of typhoid which occur it will be possible to determine approximately the proportion in which contaminated water is still to be regarded as an active source of infection, it becomes necessary to inquire whether such a proceeding will throw any real light on the subject.

The part played by water in the dissemination of such diseases as typhoid fever is so important, and the danger to public health which may arise from any weakening of faith in that mode of distribution is so considerable, that it becomes necessary to point out that however right it may be to plot out an area according to its water supply, as Dr. Seaton proposes to do, it is not by totals so collected that the matter in question can be decided. The order in which the cases occur is of quite as much importance as the place. If on comparing the incidence of typhoid fever on the different areas it should be found to be persistently greater on those deriving their water supply from one particular source that fact would, no doubt, point strongly to the probability of that source being infected. But an absence of such special incidence would say but little as to the purity of the supply. It is not by yearly returns that the water carriage of typhoid can be demonstrated, but by the association of temporary augmentations of typhoid prevalence with the occurrence of floods, especially when such irregularities in the "typhoid curve" do not occur in other areas whose water supply is derived from a different source. Every district has its own normal curve, and a distortion of that curve out of its ordinary course when floods take place is strongly suggestive of water being somehow or other concerned in the spread of the disease. The problem, however, is not so simple as may at first appear, and is complicated by the fact that a heavy rainfall often increases the ordinary local spread of typhoid just as much as it does its dissemination over large areas by a public water supply. It is then only under quite exceptional circumstances that it can ever be possible to disentangle the effects of these two modes in which excessive rainfall may increase the prevalence of typhoid fever, especially in country districts, where any floods which would be likely to foul a public supply would be still more likely to defile the local wells.

Such an exceptional set of circumstances did, however, occur in the case investigated by Mr. Shirley Murphy in regard to London, and referred to in the *Times*. In that case a quite abnormal prevalence of typhoid fever quickly followed an equally abnormal flood, and this abnormal prevalence fell upon parts of London supplied with Thames water, while those parts escaped which derived their supply from deep chalk wells or from the East London Water Company, whose works were protected by larger storage reservoirs than the other companies possessed.

Such a combination of circumstances may sometime occur within the area plotted out by Dr. Seaton, and then, if his investigations are carried out on the same lines, some definite results may perhaps be obtained if his figures should be large enough. This, however, is hardly likely to be the case. Even in London with its enormous population the number of cases of typhoid is but small, and with a scanty population the difficulties of such a statistical investigation would be much increased. It is important, however, to point out that unless the cases are plotted out chronologically as well as geographically the labour of collecting these returns will be thrown away. The evidence which has been collected during the past quarter of a century, in many countries, but more especially by the officials of our own Local Government Board, as to the water carriage of typhoid fever, is so striking and the deductions from it have been of such enormous benefit to mankind that we would not care to see it lightly put on one side, however ready we may be to admit that in the local development of the disease other forces are at work.

